# Iron Homeostasis in Tissues Is Affected during Persistent* Chlamydia pneumoniae* Infection in Mice

**DOI:** 10.1155/2017/3642301

**Published:** 2017-06-13

**Authors:** Marie Edvinsson, Jonas Tallkvist, Christina Nyström-Rosander, Nils-Gunnar Ilbäck

**Affiliations:** ^1^Department of Medical Sciences, Section of Infectious Diseases, Uppsala University, Uppsala, Sweden; ^2^Department of Biomedical Sciences and Veterinary Public Health, Swedish University of Agricultural Sciences, Uppsala, Sweden; ^3^Risk Benefit Assessment Department, National Food Agency, Uppsala, Sweden

## Abstract

*Chlamydia pneumoniae (C. pneumoniae)* may be a mediator in the pathogenesis of atherosclerosis. For its growth* C. pneumoniae* depends on iron (Fe), but how Fe changes in tissues during persistent infection or affects bacterial replication in tissues is unknown.* C. pneumoniae*-infected C57BL/6J mice were sacrificed on days 4, 8, 20, and 40. Mice had bacteria in the lungs and liver on all days. Inflammatory markers, chemokine* Cxcl2* and* interferon-gamma*, were not affected in the liver on day 40. The copper (Cu)/zinc (Zn) ratio in serum, another marker of infection/inflammation, increased on day 4 and tended to increase again on day 40. The Fe markers, transferrin receptor (TfR), Hepcidin (Hamp1), and ferroportin 1 (Fpn1), increased in the liver on day 4 and then normalized except for TfR that tended to decrease. TfR responses were similar to Fe in serum that increased on day 4 but tended to decrease thereafter. In the liver, Fe was increased on day 4 and also on day 40. The reappearing increases in Cu/Zn on day 40 concomitant with the increase in liver Fe on day 40, even though* TfR* tended to decrease, and the fact that viable* C. pneumoniae* was present in the lungs and liver may indicate the early phase of activation of recurrent infection.

## 1. Introduction

Atherosclerosis is considered an inflammatory disease [1, 2] but what causes the inflammation is not known. It has been suggested that infection with bacteria and/or viruses can contribute to the pathogenesis of atherosclerosis either via direct infection of vascular cells or via indirect effects of cytokines or acute phase proteins induced by the infection [3]. Characteristic markers of host defense reactions in common infections are increased activity in tissues of chemokines [4] and interferons [5]. Growing evidence implicates* Chlamydia pneumoniae (C. pneumoniae)*, and associated immunologic mechanisms, as an important initiator in the pathogenesis of atherosclerosis [6, 7].

*C. pneumoniae* is a respiratory pathogen that causes upper and lower respiratory tract diseases. Evidence indicates that approximately 50% of young adults and 75% of the elderly population have serological evidence of previous infection [8, 9]. Being an intracellular bacterium with a biphasic lifecycle,* C. pneumonia* alters between the metabolically inert EB (elementary body) and the metabolically active RB (reticulate body) [10]. EBs are the infectious form which are internalized by a susceptible cell and then differentiate into the metabolically active RB. RBs divide by binary fission and then differentiate into the infectious EBs that are released upon cell lysis. Under certain conditions, this cell cycle may be interrupted and instead nonreplicating “persistent bodies” are formed allowing the bacteria to maintain a chronic latent infection [10]. If* C. pneumoniae* enters into a persistent state of infection the consequence may be that it is unsusceptible to antibiotics [10]. Consequently, new treatment strategies against both acute and persistent forms of* C. pneumoniae* are needed to eradicate the bacterium from the atherosclerotic vascular wall.

Iron (Fe) is an essential element for almost all microorganisms, including* C. pneumoniae*, and increased Fe in infected tissues has, for example, been shown to promote intracellular bacterial growth of mycobacteria [11]. Accordingly, Fe restriction in cell culture inhibits growth of* C. pneumoniae* [12, 13]. The circulating peptide hormone hepcidin, produced by the liver, acts as a regulator of body Fe homeostasis [14]. The transferrin receptor (TfR) is a marker of the Fe status and together with hepcidin (Hamp 1) it regulates the ferroportin 1 (Fpn1) levels [15, 16]. During infection and inflammation hepcidin production is induced, driving a decrease in plasma Fe concentration by inhibiting absorption of Fe and promoting the sequestration of Fe in macrophages and the liver [16].

We have previously demonstrated that liver hepcidin levels in mice increase during acute* C. pneumoniae* infection and that this induction is associated with concomitantly altered Fe levels [17]. Thus, high Fe levels may not only predispose to* C. pneumonia* infection, but also could enhance the effects of* C. pneumonia* on atherogenesis. Accordingly, an increased understanding of how essential trace elements affect host defense reactions and the microorganism and how they are associated with the progression of* C. pneumoniae*-induced disease may advance prophylactic and therapeutic strategies.

Our previous study was the first to demonstrate liver hepcidin induction and associated Fe alterations during early acute* C. pneumoniae* infection [17]. We have now extended our studies to include a later stage of the disease and whether persistent colonisation of bacteria occurs and reflects a possible early phase of chronic disease. The markers we have focused on to describe Fe homeostasis are tissue changes in Fe, hepcidin, TfR, and ferroportin, and whether these changes are associated with host defense responses in terms of chemokine (Cxcl2) and interferon (IFN-*γ*) or in the Cu/Zn ratio in blood. We have previously studied the cholesterol metabolism in this* C. pneumoniae* infection and reported tissue bacterial counts and inflammatory markers Cxcl2 and IFN-*γ* in the liver until day 20 of the infection [18]. These data are also discussed in the present study together with the new and extended data in this study on day 40.

## 2. Materials and Methods

### 2.1. Mice

Adult female C57BL/6J mice (Charles River, Copenhagen, Denmark) were maintained at the Animal Department, Biomedical Centre, Uppsala, Sweden. The mice were housed at 20 ± 1°C on a 12 h light/12-h dark cycle behind strict hygienic barrier cages (TouchSLIMLine, Tecniplast, Scanbur BK A/S, Denmark) with free access to food (Labfor R36; Lantmännen, Sweden).

### 2.2. Experimental Design

The clinical isolate G-954 of* C. pneumoniae* was propagated in Hep-2 cells and stored in a sucrose-phosphate-glutamate (SPG) solution at −70°C. On day 0 of the experiment, mice aged 8–10 weeks were sedated with Fluothane (Astra Läkemedel, Södertälje, Sweden) and infected intranasally with 3 × 10^8^ ifu of* C. pneumoniae* in 30 *μ*l PBS or sham-inoculated with 30 *μ*l PBS. Groups of infected and control mice were sacrificed on days 4 (*n* = 6/group), 8 (*n* = 6/group), 20 (*n* = 5/group), and 40 (*n* = 5/group).

### 2.3. Tissue Sampling and Preparation

On each of days 4, 8, 20, and 40, infected and control mice were sedated and sacrificed. Thereafter, the thoracic cavity was opened and blood collected using heart puncture with a heparinised sterile syringe. Serum was separated from whole blood by centrifugation and stored at −70°C until further analysis. The lungs and liver were excised and aseptically divided. Tissue samples from the lungs and liver were immediately frozen at −70°C for subsequent DNA extraction. Additional tissue samples from the lungs and liver were immediately placed in RNAlater (Qiagen, Sollentuna, Sweden) overnight and then stored at −70°C for subsequent RNA extraction. Moreover, one part of the lungs was placed in 0.5 ml of SPG for subsequent culture and one part of the liver was stored at −20°C for subsequent trace element analyses.

### 2.4. Culture of* C. pneumoniae* and Confirmation of Infection

Lung tissue (approximately 30–60 mg) was collected in 500 *μ*l SPG and subsequently homogenised in the same solution with a TissueLyser (Qiagen) for 15 s on 30 Hz. Samples were then centrifuged for 10 min at 4°C at 2500 rpm. The supernatant was diluted in steps of 1 : 10 in warm cell culture medium (RPMI 1640 supplemented with 10% fetal calf serum, 20 mM HEPES (N-2-hydroxyethylpiperazine-N-2-ethanesulfonic acid), 4 mM glutamine, 20 *μ*g/ml gentamicin, and 0.05% NaHCO_3_) and 1 ml was inoculated in duplicate on confluent Hep-2 cell-layers in 24-well plates. Plates were centrifuged for 1 h at 30°C at 2270*g* and then incubated for 2 h at 35°C with 5% CO_2_. Cell culture medium was then removed and new cell culture medium supplemented with 1 *μ*g/ml cycloheximide and 40 mM glucose was added. The plates were then incubated for 72 h at 35°C with 5% CO_2_. Cells were stained with Pathfinder® Chlamydia Culture Confirmation system (Biorad, USA) according to instructions from the manufacturer and with 2 *μ*g/ml propidium iodide. The number of inclusions was counted in an inverted microscope with UV illumination. In each set of culture a negative control (SPG) and a positive control (clinical strain G-954 of* C. pneumoniae*) were included.

Approximately 10 mg of the lungs and liver were used for bacterial DNA and RNA isolations. QiaAmp DNA mini kit (Qiagen, Sollentuna, Sweden) was applied for DNA and RNeasy Fibrous Tissue kit (Qiagen, Sollentuna, Sweden) for RNA according to instructions from the manufacturers. DNA and RNA concentrations were measured using a NanoDrop ND-1000 instrument (NanoDrop Technologies Inc., Wilmington, DE, USA) and samples were subsequently stored at −70°C. Bacterial cDNA was synthesized from the RNA by using the SuperScript III First-Strand Synthesis System for RT-PCR (Invitrogen, Täby, Sweden) with random hexamers according to instructions from the manufacturer. DNA and cDNA samples were subjected to qPCR, amplifying a fragment of the* C. pneumoniae ompA* gene as previously described [19, 20].

### 2.5. Gene Expressions of Infection and Fe Metabolism Markers

Gene specific exon spanning primers to murine* Cxcl2*,* Ifng*,* Hamp1, Fpn1,* and* TfR1* were designed by the use of University of California Santa Cruz (UCSC) Genome Browser and Primer3 software. The primers were synthetized by Cybergene. The accession numbers and sequences of the primers were* Cxcl2* (NM_009140.2), 5′-TCC AGA GCT TGA GTG TGA CG-3′ (forward), and 5′-CTT TGG TTC TTC CGT TGA GG-3′ (reverse);* Ifng* (NM_008337.3), 5′-GGC CAT CAG CAA CAA CAT AA-3′ (forward), and 5′-TGA GCT CAT TGA ATG CTT GG-3′ (reverse);* Hamp1* (NM_32541), 5′-AGA AAG CAG GGC AGA CAT TG-3′ (forward), and 5′-GGG GAG GGC AGG AAT AAA TA-3′ (reverse);* Fpn1* (NM_16917.2), 5′-GTG GCA GGA GAA AAC AGG AG-3′ (forward), and 5′-TCC AAC CGG AAA TAA AAC CA-3′ (reverse) and TfR (X57349.1), 5′-CCA GTG TGG GAA CAG GTCTT-3′ (forward), and 5′-GGT ATC CCT CCA ACC ACT CA-3′ (reverse).

Quantitative gene expression was examined by RT-qPCR using a Rotor-Gene 3000 (Corbett Research) by applying One-Tube QuantiTect™SYBR®Green RT-PCR reagents (Qiagen). Expressions of target genes were normalized to the geometric average expression of the three reference genes hypoxanthine-guanine phosphoribosyltransferase 1* (Hrpt1)*, beta-2 microglobulin* (B2m),* and 18S ribosomal RNA* (18S)* as described [21]. Final primer concentration for target genes was 0.4 *μ*M and 150 ng total RNA was used as template in 25 *μ*l RT-PCR reactions. Reference genes were analyzed as described [22]. Nontemplate controls served as blanks and melt curve analysis was performed for each sample to check the specificity of the obtained PCR products. Relative quantification of the normalized mRNA expressions was performed by comparing the quantification cycle (Cq) between the tissues of the Cp-infected animals and the controls according to the 2^−(ΔΔCq)^-method [23]. Cq 40 was used as cut-off limit for detection of gene expression. Fold differences were calculated setting untreated controls to one.

### 2.6. Measurement of Trace Elements in the Liver and Serum

Tissue samples were treated as described earlier [24]. The element content was determined by inductively coupled plasma-mass spectrometry (ICP-MS; Perkin Elmer SCIEX ELAN 6000, Perkin Elmer Corp., Concord, Ontario, Canada). For quality control, every eighth sample was checked against a certified reference material: NIST bovine liver 1577a (National Institute of Standards and Technology, Gaithersburg, USA). All reference material measurements for each element were within a maximum deviation of 6% from the stated value and the maximum deviation of the precision was 5%.

### 2.7. Statistical Analyses

Statistical analyses were performed with Statistica 8.0 (StatSoft, Tulsa, OK, USA). One-Way Analysis of Variance (ANOVA) was conducted followed by Dunnett's method for multiple comparisons and the Mann–Whitney* U*-test, all considered significant at *p* < 0.05.

### 2.8. Ethics

This study was approved (C146/6) by the local Research Ethics Committee for Experimental Use at the Faculty of Medicine, Uppsala University and took into account all ethical aspects of the welfare of animals following the recommendations in “Guide for the Care and Use of Laboratory Animals” of the Swedish National Board for Laboratory Animals (CFN).

## 3. Results

### 3.1. Clinical Responses to Infection

**Figure 1:**
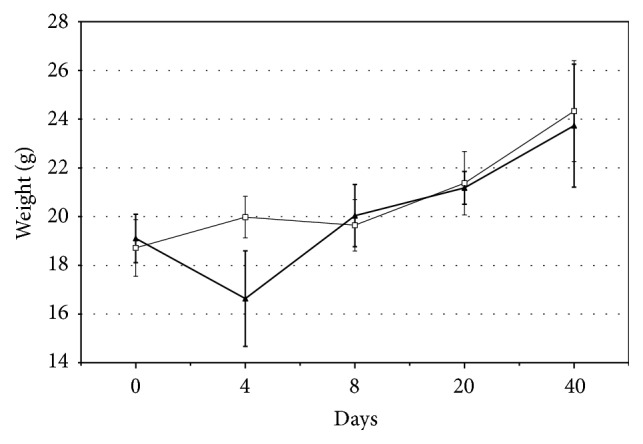
Mean body weight of* C. pneumoniae* infected (black triangles) and control mice (white boxes) during the time of the study. Whiskers extend out to the range of the standard deviation.

Mice responded to the infection with expected clinical signs of disease, including ruffled fur and inactivity, as well as by a concomitant decrease in body weight (Figure 1). Signs were most pronounced around day 4 and then gradually declined until around day 8.

### 3.2. Detection of* C. pneumoniae* in the Lungs and Liver

*C. pneumoniae* was isolated from the lungs by culture in Hep-2 cells on each of the sampling days and bacterial DNA in the lungs was demonstrated on all days. mRNA from* C. pneumoniae* was demonstrated in the lungs on days 4 and 8. In the liver* C. pneumoniae* DNA was detected on all days but mRNA was only detected in 1/6 mice on days 4 and 8. The number of mice positive on all days is shown in Table 1. Data on days 4, 8, and 20 have previously been published in another study with a different focus on* C. pneumonia* infection [18].

### 3.3. Immune Markers of Inflammation/Infection

**Figure 2:**
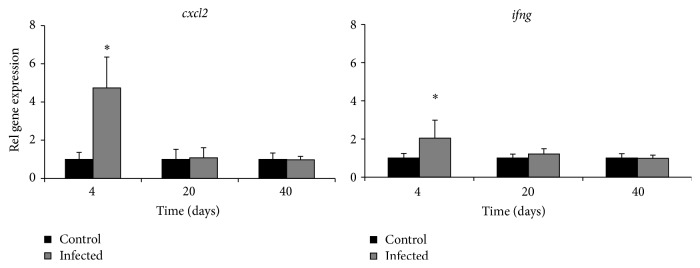
Relative gene expression of the chemokine Cxcl2 and IFN-*γ* in the liver of control mice (black bars) and* C. pneumoniae* infected mice (grey bars) on days 4 (*n* = 6), 20 (*n* = 5) and 40 (*n* = 5) of the infection. Columns represent mean values with whiskers extending to the standard deviation. Asterisks denote a significant difference (^*∗*^*p* < 0.05) between infected and control mice.

The gene expressions of Cxcl2 and IFN-*γ* were measured in the liver on day 40 (Figure 2). Both these markers have previously been shown to be greatly increased on day 4 but then on day 20 in principle normalized [18]. Accordingly, in the present study on day 40 both IFN-*γ* and Cxcl2 were unaffected by the infection.

### 3.4. Markers Related to Fe Metabolism

**Figure 3:**
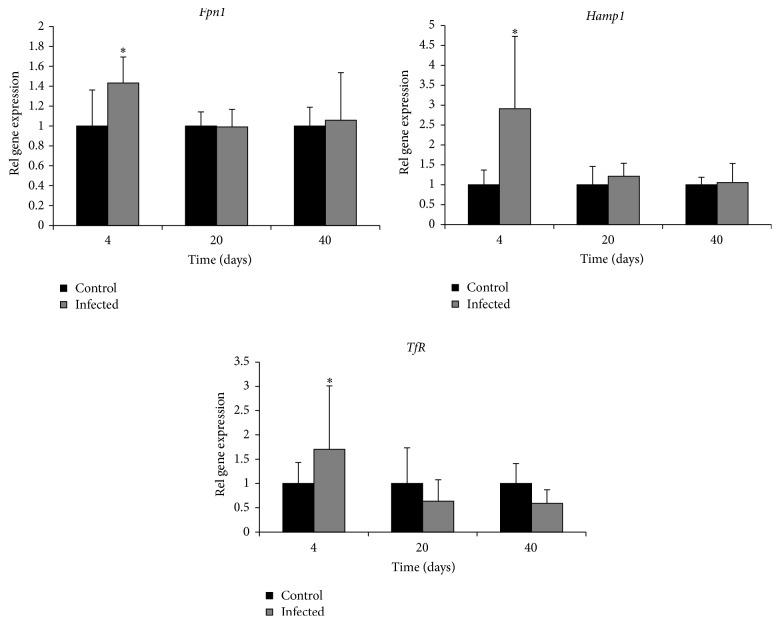
Relative gene expression of the Fe markers ferroportin (Fpn1), hepcidin (Hamp1), and transferrin receptor (TfR) in the liver of control mice (black bars) and* C. pneumoniae* infected mice (grey bars) on days 4 (*n* = 6), 20 (*n* = 5), and 40 (*n* = 5) of the infection. Columns represent mean values with whiskers extending to the standard deviation. Asterisks denote a significant difference (^*∗*^*p* < 0.05) between infected and control mice.

Gene expressions of Hamp1, Fpn1, and TfR were upregulated 3.5-, 1.5-, and 1.7-fold, respectively, in the livers of the infected animals on day 4, whereas no statistically significant effects were observed on days 20 or 40 (Figure 3).

### 3.5. Trace Elements

**Figure 4:**
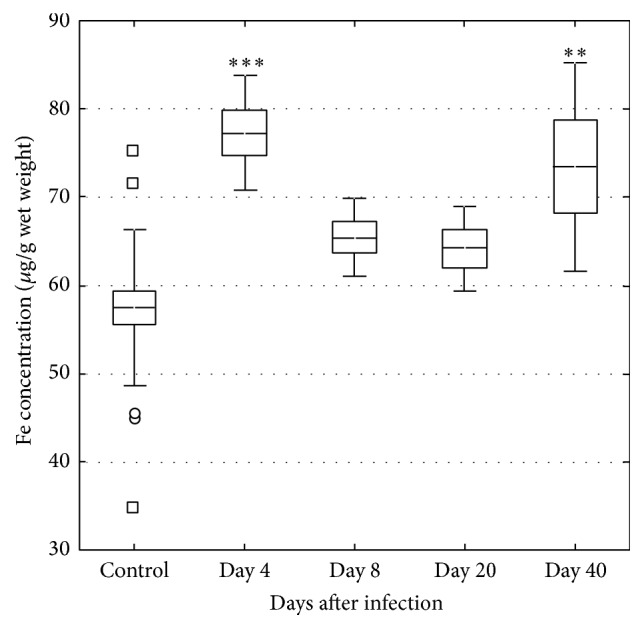
Concentration of Fe in the liver in control (*n* = 22) and* C. pneumoniae* infected mice on days 4 (*n* = 6), 8 (*n* = 6), 20 (*n* = 5), and 40 (*n* = 5). The central line indicates the mean value, the box is limited by the mean value ± standard error, and the whiskers extend out to the range of the standard deviation. Asterisks denote a significant difference (^*∗∗*^*p* < 0.01, ^*∗∗∗*^*p* < 0.001) between infected and control mice.

**Figure 5:**
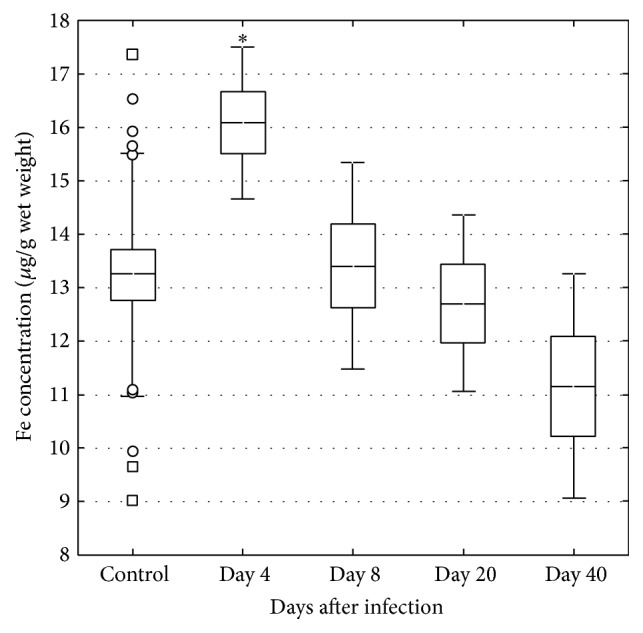
Concentration of Fe in the serum in control (*n* = 22) and* C. pneumoniae* infected mice on days 4 (*n* = 6), 8 (*n* = 6), 20 (*n* = 5), and 40 (*n* = 5). The central line indicates the mean value, the box is limited by the mean value ± standard error, and the whiskers extend out to the range of the standard deviation. Asterisks denote a significant difference (^*∗*^*p* < 0.05) between infected and control mice.

**Figure 6:**
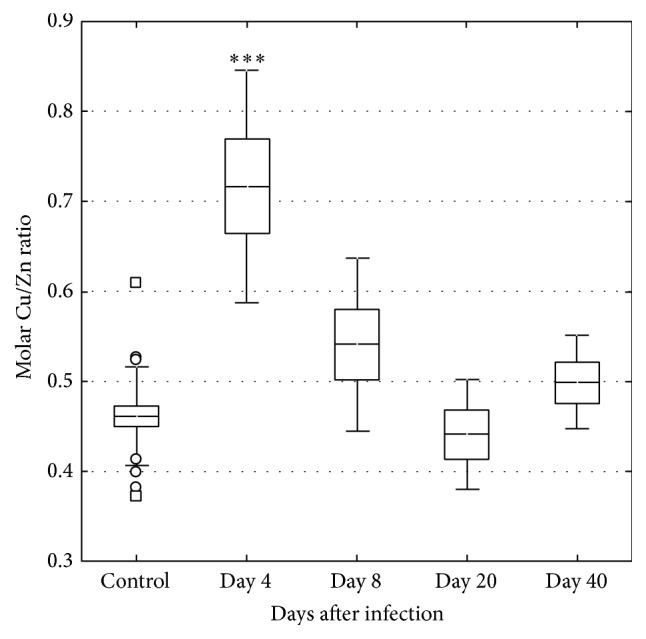
The molar Cu/Zn ratio in serum in control (*n* = 22) and* C. pneumoniae* infected mice on days 4 (*n* = 6), 8 (*n* = 6), 20 (*n* = 5), and 40 (*n* = 5). The central line indicates the mean value, the box is limited by the mean value ± standard error, and the whiskers extend out to the range of the standard deviation. Asterisks denote a significant difference (^*∗∗∗*^*p* < 0.001) between infected and control mice.

Fe levels increased in the liver on days 4 and 40 (Figure 4). Fe increased in serum on day 4 but then tended to decrease during the course of the infection (Figure 5). The molar Cu/Zn ratio in serum increased on day 4 but then returned to levels within the normal range, however with an indication of again increasing level on day 40 (Figure 6).

## 4. Discussion

In the present study Fe levels increased in serum and liver on day 4, corresponding to the early acute phase of the* C. pneumoniae* infection. At this time point, there is according to previous data a greatly increased gene expression of the immune markers Cxcl2 and IFN-*γ* [18]. A corresponding increase was observed in the present study in in markers related to Fe metabolism, that is, hepcidin, ferroportin 1, and TfR in the liver. Between days 4 and 20, mice recovered from the infection in terms of weight changes and clinical symptoms, as well as in changes associated with inflammatory processes in the liver in terms of the Cu/Zn ratio and previous data on Cxcl2 and IFN-*γ* [18]. However, on day 40 a majority of mice were still positive for* C. pneumoniae* concomitant to a tendency of an increased Cu/Zn ratio in serum, but no tendency of inflammatory responses in the liver in terms of Cxcl2 and IFN-*γ*. This is a notable finding together with the observed mobilization of Fe into the liver even though the gene expression of liver TfR indicated downregulation of the need for Fe. These changes on day 40 in Fe status and the concomitant presence of bacteria in tissues may reflect the early phase of reactivation of a potentially chronic* C. pneumoniae* infection, but at a subclinical level without pronounced inflammatory processes in the tissue.

We [17] and others [25] have demonstrated that* C. pneumoniae* disseminates to and can be metabolically active in the liver. Presence of* C. pneumoniae* in the liver may influence the metabolism and the trace element balance in the body. In the present study* C. pneumoniae* DNA was demonstrated in the liver even on day 40. Presence of DNA indicates viable bacteria in that heat-inactivated bacteria are known to be rapidly degraded in mice [26]. Accordingly, in the present study no bacterial mRNA was demonstrated after day 8, indicating that bacteria found after day 8 probably are in a nonmetabolically persistent state. However, they can evidently still induce immunological responses as reflected by the tendency of an increase in the Cu/Zn ratio on day 40.

Elevated body Fe stores have been considered a risk factor for cardiovascular diseases [27–29] and analyses of atherosclerotic plaques from patients undergoing carotid endarterectomy have shown elevated levels of Fe [30]. During experimental infections, there is generally a decrease in serum Fe, which is due to an uptake of Fe in the liver concomitant with a decrease in intestinal Fe absorption [31]. In the present study Fe in serum and gene expression data of hepcidin, transferrin 1, and TfR, reflecting Fe status in the liver, showed an early increase that tended to decrease during the course of the infection. This observation is in contrast to a previous study where Fe showed an earlier decrease [17]. An increase in hepcidin in response to infectious or inflammatory stimuli seems to be responsible for the characteristic hypoferraemia of inflammation, which develops within a few hours of a systemic infection [16]. Accordingly, in the present study Fe in serum decreased over time and concomitantly increased in the liver.

During infections, intestinal Zn absorption is increased [31], but it is rapidly taken up by the liver, resulting in decreased plasma levels of Zn [32]. In contrast, Cu levels in plasma increased in the present study, probably because of the increased synthesis and release of ceruloplasmin [31], which is a copper-binding plasma protein, as well as an acute phase reactant [32]. An increase in the Cu/Zn ratio has therefore been used as a marker of infection/inflammation [33] and to indicate infection before development of clinical signs [34]. Moreover, epidemiological studies suggest that an increased Cu/Zn ratio is associated with a higher risk of cardiovascular mortality [35–37]. During the acute phase of a previous experimental* C. pneumoniae* infection in mice, the serum Cu/Zn ratio showed a significant increase [17]. A similar and transient response was also demonstrated on day 4 in the present study when clinical signs tended to be most severe and as shown in a previous study the gene expressions of the immune markers Cxcl2 and IFN-*γ* are greatly increased [18]. Notable in this study was that the Cu/Zn ratio again tended to increase on day 40, but without detectable immune responses in the liver in terms of Cxcl2 and IFN-*γ*. The presence of bacteria in the liver was concomitantly associated with Fe mobilization even though TfR was downregulated. In addition, viable and active bacteria were also still present in the lungs. Accordingly, our research team has demonstrated elevated levels of Fe in sclerotic aortic heart valves [38], where a large portion of the valves also were positive for* C. pneumoniae* and these patients also had an elevated Cu/Zn ratio in serum [39]. Furthermore, an increased Cu/Zn ratio has also been demonstrated in patients with thoracic aortic aneurysm (where 26% were positive for* C. pneumoniae* in the aneurysm) [40] as well as in patients with bacteremia [41].

Fe can be looked upon as a double edge sword. Firstly, Fe modulates host defense as it in macrophages regulates their cytokine production [16]. Secondly, microorganisms that enter the host face multiple mechanisms that restrict their ability to obtain Fe and thereby limit their pathogenicity [42]. These hepcidin-mediated effects are causing a decrease in plasma Fe concentration but sequestration of Fe in macrophages and the liver [16]. Consequently, it is reasonable to assume that the redistribution of Fe in tissues during infection is a normal host response partly aiming to deprive pathogens of essential elements [31]. The control of Fe homeostasis seems vital to the course of the infection and withholding the metal from microbes has been proven to be an efficient strategy for infection control [43]. For example, hepcidin, produced by the liver and regulating Fe homeostasis [14], is increased by acute* C. pneumoniae *infection [17].* Hepcidin*, as well as* ferroportin 1* and* TfR*, was also increased in the early phase of the present infection. Increased* hepcidin *levels result in reduced Fe efflux from hepatocytes and macrophages [15]. In the present study Fe concentration in the liver was increased on day 4 when clinical signs were most severe. Furthermore, the Fe content in the liver was again increased on day 40, regardless of a decrease in TfR expression and a concomitant tendency of again increasing Cu/Zn ratio. However, Fe is an essential element for chlamydial growth [13] and suppression of chlamydial growth and infectivity in epithelial cells have been shown when artificial Fe starvation is induced by Fe-chelating agents [44]. Furthermore, in mice Fe challenge was associated with a higher bacterial growth in peritoneal macrophages [45] and Fe supplementation to endothelial cells induced proliferation of* C. pneumoniae* [46]. Consequently, based on our results and other published data, it is reasonable to speculate that hepcidin-induced Fe sequestration in macrophages paradoxically enhances Fe availability for* C. pneumoniae* that occupy this intracellular niche [16, 42]. A consequence of this could be less efficient eradication of the bacteria and development of chronic disease.

*C. pneumoniae* is disseminated in the body by circulating monocytes [26] and a recent clinical study demonstrated elevated Fe concentrations in the labile Fe pool in circulating monocytes in patients with cardiovascular disease [47]. Furthermore, patients with high body Fe stores have an increased risk of developing acute myocardial infarction [48]. The exact mechanism by which Fe influences the atherosclerotic process is not yet clarified but is believed to involve oxidative modification of important elements of the atherosclerotic process such as low-density lipoprotein (LDL) [48]. However, another contributing mechanism may involve the intracellular bacterium* C. pneumoniae* whose growth depends on Fe [12]. The elevated body Fe stores and increased Fe concentration in macrophages may promote the activation of* C. pneumoniae* resulting in increased inflammation and stimulation of the atherosclerotic process. Clinical studies have demonstrated that a decrease of body Fe store proved beneficial on cardiovascular outcome if performed before the age of 60 [49]. This reduction of body Fe stores may also have an impact on* C. pneumoniae* infection as restriction of Fe in cell cultures of* C. pneumoniae *reduced bacterial growth [12]. However, whether this would also be the case in humans remain to be investigated.

In the present and in our previous study [17] Fe levels in serum decreased during the course of the* C. pneumoniae* infection. Thus, persistence of* C. pneumoniae* in the liver and a decreased expression of TfR in the liver but concomitant increase of Fe on day 40 in the liver but not in serum were notable. These findings, when taken together with the pattern of changes in the Cu/Zn ratio, may indicate a general low-grade inflammation and/or increased bacterial activity and recurrence of the infection, but in a subclinical and chronic state since the mice showed no clinical sign of disease.

## Figures and Tables

**Table 1:** Numbers of infected mice positive for *C. pneumoniae* in the lungs and liver with qPCR (DNA), qPCR (mRNA) or culture. Total number of animals infected on days 4, 8, 20, and 40 was 6, 6, 5, and 5, respectively.

Tissue and method	Day 4	Day 8	Day 20	Day 40
Lung culture	5/6	6/6	3/5	3/5
Lung qPCR (DNA)	6/6	6/6	3/5	4/5
Lung qPCR (mRNA)	3/6	4/6	0/5	0/5
Liver qPCR (DNA)	4/6	3/6	1/5	1/5
Liver qPCR (mRNA)	1/6	1/6	0/5	0/5
